# Silver-Silica nanoparticles induced dose-dependent modulation of histopathological, immunohistochemical, ultrastructural, proinflammatory, and immune status of broiler chickens

**DOI:** 10.1186/s12917-022-03459-2

**Published:** 2022-10-04

**Authors:** Asmaa F. Khafaga, Moustafa M. G. Fouda, Ali B. Alwan, Nader R. Abdelsalam, Ayman E. Taha, Mustafa S. Atta, Waleed M. Dosoky

**Affiliations:** 1 Department of Pathology, Faculty of Veterinary Medicine, Alexandria University, Edfina, 22758 Egypt; 2 Pretreatment and Finishing of Cellulose Based Textiles, Textile Research and Technology Institute (TRT), National Research Center, 33 El-Buhouth St, Dokki, Giza, 12311 Egypt; 3 Agricultural Botany Department, Faculty of Agriculture (Saba Basha), Alexandria University, Alexandria, 21531 Egypt; 4 Department of Animal Husbandry and Animal Wealth Development, Faculty of Veterinary Medicine, Alexandria University, Edfina, 22758 Egypt; 5 Physiology Department, Faculty of Veterinary Medicine, Kafrelsheikh University, Kafrelsheikh, 33516 Egypt; 6 Department of Animal and Fish Production, Faculty of Agriculture (Saba Basha), Alexandria University, Alexandria, 21531 Egypt

**Keywords:** AgNPs, Broiler chicken, Immunoglobulins, CD45, Histopathology, Ultrastructural morphology, Proinflammatory cytokine

## Abstract

Silver nanoparticles (AgNPs) are a powerful disinfectant, but little information is available on their potential use as a growth promoter and the safety margin of this. In this study, 480 1-day-old Cobb chicks were assigned to one control and three treated groups. The treated groups were supplemented with silver-doped silica nanoparticles (SiO_2_@AgNPs) at three dietary levels (8, 16, and 20 mg/kg diet) for 35 days. The results revealed no significant changes in the growth performance and oxidative parameters, and in most of the hematological and biochemical parameters among the control and treated groups. In contrast, dose-dependent adverse effects were exerted on the histopathological structure and immunohistochemical expression of CD45 in liver, kidneys, and lymphoid organs (spleen, bursa, and thymus). In addition, the relative weight of lymphoid organs and the serum levels of immunoglobulins M and G were significantly diminished. Moreover, the gene expression of proinflammatory cytokines (IL-β1 and TNF-α) and the ultrastructural morphology in breast muscle showed significant dose-dependent alterations. It could be concluded that the dietary supplementation of SiO_2_@AgNPs at a level of 8 mg/kg diet or more has dose-dependent proinflammatory and immunosuppressive effects on broiler chickens.

**Supplementary Information:**

The online version contains supplementary material available at 10.1186/s12917-022-03459-2.

## Introduction

The technology of nanoparticles is currently used in various applications, such as in the chemical industry, biomedicine, nutrition, and medication [1]. The term “nanoparticles” refers to particles of less than 100 nm in size that exhibit dramatically modified physical and chemical properties compared with the normal elements. They can easily penetrate into tissues and cross cell membranes due to their small size [2–4].

Silver nanoparticles (AgNPs) have physical and chemical properties that are distinct from those of their larger counterparts due to their chemical stability and very large surface-to-volume ratio [5–8]. Nanosilver solution is composed of colloidal suspensions of silver ions, which is more stable than other solutions. Aqueous solutions carrying silver nanoparticles embedded in various media, such as silica or polymers, are considered as being among the most powerful disinfectants [9–12]. Importantly, AgNPs can affect many microorganisms, such as viruses, bacteria (gram-positive and -negative), molds, and fungi, having potent germicidal effects on up to 650 different types of bacteria [13]. Specifically, their use for treating many types of pathogen, including bacteria, fungi, and viruses such as influenza and Newcastle, has been recommended [14].

The European Food Safety Authority (EFSA) does not raise safety concern for the consumer if used silver nanoparticles as an additive at up to 0.025% w/w in polymers [15]. This is because of the migration of silver occurs up to 6 μg silver/kg food in soluble ionic form from the surface of the additive particles, which is below the group restriction of 50 μg silver/kg food proposed by the Scientific Panel on Food Additives, Flavorings, Processing Aids and Materials in Contact with Food in 2004 [15]

Recently, AgNPs have also played a role in animal and specifically poultry nutrition as anti-inflammatory and immunostimulatory agents [15], improving the growth performance of broiler chickens [16]. A few studies on the use of AgNPs in rearing chickens have been performed [17–19]; however, the obtained findings are conflicting and do not explicitly indicate whether AgNPs might be safely supplied to poultry. The use of nanoparticles of various sizes, characteristics, and embedding media in these experiments may explain the discrepant findings.

Various research groups have conducted in vivo experiments using low levels of AgNPs; they concluded that AgNPs did not have adverse effects on embryonic growth, DNA structure [7, 9, 20], or the immune system [21, 22]. In addition, [23] clarified that AgNPs (at concentrations of 0.1, 0.5, and 1.0%) have non-cytotoxic effects on cell lines in vitro. In contrast, it has been proven that, at concentrations of 2.5–50 μg/mL, AgNPs have cytotoxic effects on human mesenchymal stem cells [24] and on four different mammalian cell lines [25].

Concerning the inflammatory activity of AgNPs, a few studies have illustrated their proinflammatory and immunosuppressive effects via inducing the regeneration of reactive oxygen species (ROS) [26, 27]. In contrast, others [28, 29] have outlined their potent anti-inflammatory properties. It is well established that colloidal silver nanoparticles are small enough to infiltrate cells and then nuclei, where they associate directly with nuclear DNA, resulting in modulation of the in vivo and in vitro gene expression profiles [6, 28, 30], especially for inflammatory response-related genes such as IL-1β and TNF-α [31].

Limited evidence is available on the safe usage of AgNPs administered in the diet in poultry production. However, our recently reported study proved that the safety margin of orally applied silver-doped silica nanoparticles (SiO_2_@AgNPs) in poultry production is 4 mg/kg diet. However, mild adverse effects were reported at a dose of 8 mg/kg diet. Therefore, in this study, we hypothesized that the application of SiO_2_@AgNPs to poultry in the diet at higher levels may exert adverse effects on the treated chickens. The objective of this study was to evaluate the effect of the dietary application of different doses of SiO_2_@AgNPs on growth performance and biochemical, hematological, immunological, and oxidative parameters. In addition, the histopathological and immunohistochemical findings of liver, kidney, spleen, thymus, and the bursa of Fabricius, as well as liver residue levels, were investigated. Moreover, the gene expression of IL-1β and TNF-α and the ultrastructural morphology of chicken breast muscle were also evaluated.

## Materials and method

### Ethics statement

The experimental procedure was performed in accordance with the Institutional Guidelines rules for the Care and Use of Laboratory Animal and with the ARRIVE guidelines. The experiment was approved by the Ethics Committee of the Faculty of Agriculture (Saba Basha), Alexandria University.

### Preparation of silver-doped silica nanoparticles (SiO2@AgNPs) in powder form

Silver-doped silica nanoparticles (SiO_2_@AgNPs) were prepared in powder form, using starch, via chemical reduction and sol–gel technique, as we previously described. The materials used, detailed method of preparation, and full transmission electron microscopy (TEM) characterization are presented and linked as supplementary materials (S1).

### Experimental design

The current experiment was performed at the Faculty of Agriculture (Saba Basha), Department of Animal and Fish Production, Alexandria University, from June to July 2020. A total of 480 1-day-old Cobb male chicks (with an average weight of 42.6 g) were obtained from a commercial local hatchery. The chicks were allocated into 24 pens (1.35 × 1.45 m) at random. The pens were placed in an open-sided house and used for experiments with the following design: 4 treatments × 6 replicates × 20 chicks. During the experiments, the humidity ranged from 55 to 75% and the temperature was adjusted to 33 °C at day 1 of age, followed by a gradual decrease to 31 °C after 14 days of age. The chicks were exposed to an intermittent lighting system (3 h light:1 h dark) and reared in wire batteries under similar hygienic, management, and environmental conditions. At day 7 of age, the chicks were vaccinated with HB1 against Newcastle disease (ND) and infectious bronchitis and with La-Sota strain against ND at day 21. The chicks had free access to food and water. They were fed on a starter ration (crude protein 22.98%, yellow corn 55.75%, and soybean 38%) from day 1 to day 21 of age, and a growing ration (crude protein 20.98%, yellow corn 59.59%, and soybean 33.15%) from day 22 to the end of the experimental period (35 days of age). The experimental diet was formulated in accordance with [32].

Chicks received the dietary treatments as follows: Group 1, chicks were fed on basal diets and served as a control; and Groups 2, 3, and 4, chicks were fed on basal diets supplemented with 8, 16, and 20 mg of SiO_2_@AgNPs/kg diet, respectively. The experimental period lasted 35 days.

### Estimation of growth performance parameters

At day 35 of age, the chicks’ live body weight (LBW), body weight gain (BWG), feed consumption, and feed conversion ratio (g feed/g gain) were recorded. No mortality occurred during the experiment.

### Evaluation of hematological, biochemical, and antioxidative parameters

At day 35 of age, 18 chicks were randomly selected from each treated group (3 birds/ replicate) and slaughtered. From each bird, two blood samples were immediately collected from the brachial vein. The first sample was collected on heparin as anticoagulant (0.1 ml of heparin to 1 ml of blood) and used for a hematological count. The second sample was collected without anticoagulant for serum preparation. The collected serum samples were centrifuged for 15 min at 3500 rpm and stored at − 18 °C for further estimation of the protein profile [total protein (TP) and albumin (ALB)], liver function [alanine aminotransferase, aspartate aminotransferase (AST), and alkaline phosphatase], kidney function (uric acid and creatinine), minerals (calcium and phosphorus), lipid profiles (total lipids, cholesterol, low-density lipoprotein, high-density lipoprotein, and triglycerides), and oxidative parameters [malondialdehyde (MDA), total antioxidant capacity (TAC), and catalase (CAT)]. All parameters were estimated using commercially available kits from Biodiagnostic, Giza, Egypt (www.bio-diagnostic.com).

### Evaluation of immunological parameters

Serum immunoglobulin (Ig) fractions were estimated as previously described by [33]. Phagocytic activity was evaluated in accordance with the method of [34]; briefly, 50 µg of Candida albicans culture was added to 1 ml of citrated blood samples collected from control and treated chicks, and shaken for 3–5 h in a water bath (23–25 °C). Blood films were prepared and stained with Giemsa stain. Phagocytosis was calculated by estimating the proportion of macrophages engulfing yeast cells among 300 randomly counted phagocytes and represented as the percentage of phagocytic activity (PA) (PA = percentage of phagocytic cells containing yeast cells). Phagocytic index (PI) was estimated as the number of engulfed organisms in the phagocytic cells.

$$\mathrm{Phagocytic}\;\mathrm{index}\;\left(\mathrm{PI}\right)=\frac{\mathrm{Number}\;\mathrm{of}\;\mathrm{yeastcells}\;\mathrm{phagocytized}}{\mathrm{Number}\;\mathrm{of}\;\mathrm{phagocytic}\;\mathrm{cells}}$$

To determine the humoral–antibody titer against Newcastle disease virus (NDV), blood samples were collected at the end of the experiment (14 days after La-Sota strain vaccination). Serum samples were prepared by centrifugation for 15 min at 4000 rpm and used to perform the hemagglutination–inhibition test (HI).

### Lymphoid organ weight and carcass traits

At 35 days of age, six chicks were selected at random from each treatment and fasted for 12 h. Then, they were weighed, slaughtered, allowed to bleed out completely, and weighed again to estimate the relative weight of immune-related organs (thymus, spleen, and bursa).

### mRNA expression of interleukin 1 beta (Il-1β) and tumor necrosis factor alpha (TNF-α) in muscle tissues

The mRNA expression of IL-1β and TNF-α was determined by RT-PCR. Briefly, TRIzol reagent (Invitrogen, Life Technology, Carlsbad, CA, USA) and Quantification Nanodrop were used for the extraction of about 100 mg of total RNA of breast muscle tissue. DNA synthesis was performed using a cDNA synthesis kit (Fermentas, Waltham, MA, USA); samples of A_260_ or higher A_260_/A_280_ RNA were used. The amplification of cDNA, a household-gene mix has been included with SYBR Green, and the list of primers is presented in Table 1 and GAPDH. Data were analyzed by the 2.^−ΔΔT^ method [35]

**Table 1:** Primer sequences for the genes

	Forward	Reverse	Accession number
B-actin	GTCCACCTTCCAGCAGATGT	ATAAAGCCATGCCAATCTCG	396,526
IL1B	AGGTGAGAGTCCCGAGTCC	GTAGGTGGCGATGTTGACCT	AJ245728
TNFα	CAGGACAGCCTATGCCAACA	AACTCATCTGAACTGGGCGG	HQ739087

### Histopathological evaluation

After slaughter, small samples (1 × 2 cm) of liver, kidneys, thymus, bursa, and spleen were immediately collected from the control and treated chicks. The collected samples were washed and immersed in neutral-buffered formalin solution (10%) for at least 48 h for fixation. The fixed samples were prepared through the routine paraffin-embedding technique [36]. Several 4-μm-thick sections were prepared and routinely stained with hematoxylin and eosin. Blinded evaluation and image capturing were performed by an experienced pathologist (AFK). Representative micrographs were captured with a digital camera (Leica EC3; Leica, Germany) connected to a microscope (Leica DM500).

### Immunohistochemical evaluation

Four-μm-thick sections were prepared from each paraffin block, deparaffinized, rehydrated in a decreasing concentrations of ethanol, and retrieved for antigens using citrate-buffered saline (0.01 mol/L; pH 6.0). After that, the depletion of endogenous peroxidase activity was carried out via H_2_O_2_ in phosphate-buffered saline [0.3% (v/v)]. The nonspecific immunological reaction was blocked through the incubation of samples with 10% (v/v) normal goat serum for 60 min. Next, overnight incubation of prepared sections with mouse anti-chicken monoclonal CD45 (MCA2413GA; Bio-Rad Laboratory, Athens, Greece) was performed at 4 °C. Sections were washed with phosphate-buffered saline (PBS), incubated with biotin-conjugated goat anti-mouse IgG antiserum (Histofine kit; Nichirei Corporation, Japan) for 1 h, rewashed with PBS, and re-incubated with streptavidin–peroxidase conjugate (Histofine kit; Nichirei Corporation, Japan) for 30 min. The streptavidin–biotin reaction was visualized via 3,3′-diaminobenzidine tetrahydrochloride (DAB)–H_2_O_2_ solution (pH 7.0, for 3 min). Finally, Mayer’s hematoxylin counterstaining was performed.

### Ultrastructural evaluation of muscle tissues

Immediately after slaughter, small specimens were obtained from breast muscle. Specimens were cut into small pieces (about 1 mm^3^) and immediately fixed in 3% glutaraldehyde solution (Merck, Darmstadt, Germany) for at least 3 h in 0.1 M PBS (pH 7). Fixed samples were washed in two changes of buffer and transferred to a 1% osmium tetroxide solution (Electron Microscope Science, Sigma-Aldrich) for 60 min in 0.1 M PBS (pH 6.9). Then, samples were rewashed in 0.1 M PBS for 5 min, dehydrated in increasing concentrations of ethanol, and impregnated with Epon embedding resin. Samples were embedded at 60 °C for 48 h and blocked. Semi-thin sections were prepared and stained with 1% basic Toluidine blue for light microscopy examination. After that, ultrathin Sects. (50–80 nm) were processed from the selected areas and placed onto copper grids (200 mesh). Finally, section contrasting was performed using uranyl acetate dihydrate (2%) and lead citrate. Tissues were examined and captured by JEM-1220 TEM (JEOL, Tokyo, Japan), with a Morada 11-megapixel camera (Olympus Soft Imaging Solutions GmbH, Münster, Germany).

### Evaluation of tissue residue

To evaluate SiO_2_@AgNP residue in liver tissues, 2 g of hepatic tissue was collected and digested in 5 mL of HNO_3_ and 2 mL of H_2_SO_4_ solution at 60 °C for 30 min. After that, the tube was cooled and 10 ml of conc. HNO_3_ was added to it and heated slowly up to 120 °C. H_2_O_2_ was then gradually added until the tube’s solution became clear. The sample was heated again and cooled. The residual remains in the tube were then dissolved in 100 mL of ultra-deionized water. The amount of SiO_2_@AgNPs in diluted solution was determined using ICP-OES (Model iCAP 7400 Duo; Shanghai, China).

### Statistical analysis

Data were collected, computed, and statistically analyzed via one-way ANOVA using GLM procedures, with the aid of statistical analysis software [37]. The differences between means of control and treated groups were determined using the Student–Newman–Keuls test. Data are provided as mean and standard error (SEM). Values were considered statistically significant at *p* < 0.05.

## Results

### Characterization of silver-doped silica nanoparticles (SiO2@AgNPs) in powder form

Characterization of SiO_2_@AgNPs was performed via the transmissible electron microscope (TEM). The average hydrodynamic diameter of SiO_2_@AgNPs is 51 nm; where they had negative zeta potential (-33 mv) due to the presence of stabilizing agent. The scanning electron microscope (SEM) was used to identify the morphology and surface structure of SiO_2_@AgNPs. Nanoparticles appeared as fairly uniform spherical particles with an average size of 150–250 nm. The full characterization of SiO_2_@AgNPs was supplied as supplementary materials (S1).

### Evaluation of growth performance parameters

As shown in Table 2, no significant changes were identified for LBW, BWG, feed consumption (g/bird) and feed conversion ratio of the boiler chickens in the different experimental groups at 21 and 35 days of age.

**Table 2:** Effect of different levels of silver doped silica nanoparticles (SiO_2_@AgNPs) on productive performance of boiler chickens from 1–35 days of age

Traits	Silica- silver nanoparticles levels (mg/kg diet)					
	0	8	16	20	SEM	*P* value
Body weight (g)						
1 day	42.73	42.44	42.98	42.42	0.30	0.916
21 days	881.66	890.54	893.84	900.28	7.07	0.830
35 days	1919.30	1953.10	1927.00	1958.50	17.38	0.828
Body weight gain (g)						
1–21 days	838.93	848.02	850.87	857.35	7.03	0.832
21–35 days	1037.60	1062.60	1033.20	1059.00	14.23	0.872
1–35 days	1876.60	1910.66	1884.00	1916.40	17.41	0.822
Feed consumption (g/bird)						
1–21 days	1107.10	1120.9	1125.00	1138.50	7.22	0.496
21–35 days	1533.10	1531.4	1523.60	1535.00	6.26	0.857
1–35 days	2640.20	2645.6	2648.60	2673.60	10.15	0.733
Feed conversion ratio (g feed/g weight gain)						
1–21 days	1.32	1.32	1.32	1.33	0.01	0.916
21–35 days	1.49	1.44	1.49	1.45	0.02	0.877
1–35 days	1.41	1.38	1.41	1.40	0.01	0.958

### Evaluation of hematological parameters

The data in Table 3 reveal no significant variation in RBC count and PCV % among the different groups. In contrast, a significant (*P* = 0.014) increase in WBC count and a significant (*P* = 0.053) reduction in Hb (g/dl) were reported in chickens feed 20 mg of SiO_2_@AgNPs, compared with the control group. In addition, the proportions of lymphocytes (L), heterophils (H), H/L ratio, monocytes, basophils, and eosinophils showed no significant differences between different groups compared to controls.

**Table 3:** Effect of different levels of silver doped silica nanoparticles (SiO_2_@AgNPs) on some hematological and immunological parameters of boiler chickens at 35 days of age

Items	Silica- silver nanoparticles levels (mg/kg diet)					
	0	8	16	20	SEM	*P* value
Hematological parameters						
Red blood cells (RBCs 10^6^/mm^3^)	1.57	1.41	1.53	1.47	0.030	0.396
White blood cells (WBCs 10^3^/mm^3^)	21.00^b^	20.77^b^	22.67^ab^	24.00^a^	0.39	0.014
Hemoglobin (Hb g/dl)	10.67^a^	10.55^a^	10.00^ab^	9.67^b^	0.17	0.053
Packed cell volume (PCV %)	33.67	34.22	32.00	32.33	0.32	0.051
Lymphocytes (%)	62.33	61.31	63.33	63.00	0.86	0.650
Heterophils (%)	32.70	32.98	31.33	31.67	1.04	0.851
H/L ratio	1.92	2.00	2.14	2.06	0.25	0.611
Monocytes (%)	3.00	3.35	3.67	4.00	0.33	0.782
Basophils (%)	0.67	0.77	1.00	0.67	0.10	0.590
Eosinophils (%)	1.30	1.26	0.67	0.67	0.19	0.426
Immunity parameters						
Phagocytic activity (PA)	19.33	19.95	21.00	21.00	0.35	0.253
Phagocytic index (PI %)	1.97	1.93	1.93	2.03	0.32	0.798
Antibody titter against NDV; HI	5.67	6.01	6.00	6.33	0.19	0.678
Immunoglobulin M; IgM (mg/dl)	23.53^a^	23.46^ab^	23.13^c^	23.23^bc^	0.06	0.019
Immunoglobulin G; IgG (mg/dl)	973.67^a^	970.12^ab^	965.00^c^	968.00^bc^	0.98	0.001
Lymphoid organs weight (%)						
Spleen	0.14^a^	0.08^b^	0.09^b^	0.07^b^	0.01	0.017
Bursa	0.09	0.10	0.13	0.16	0.01	0.189
Thymus	0.19^b^	0.30^a^	0.33^a^	0.35^a^	0.02	0.001

^a-c^ Means in the same row having different letters are significantly different

### Evaluation of lymphoid organ relative weight and immunological parameters

Concerning the relative weight of lymphoid organs, no significant difference was reported for the bursa of Fabricius among the different treatments. However, a significant increase (*P* = 0.001) in the relative weight of the thymus and a significant reduction (*P* = 0.017) in the relative weight of the spleen were reported in all treated groups compared with the control treatment (Table 3).

Furthermore, there were no significant differences in antibody titer against NDV, PA, and PI% in all treated groups compared with the control group. However, significant decreases in IgM (*P* = 0.019) and IgG (*P* = 0.001) were noted in chickens supplemented with 16 and 20 mg of SiO_2_@AgNPs compared with control chickens (Table 3).

### Evaluation of serum biochemical and oxidative parameters

As illustrated in Table 4, significant increases in globulin (g/dl; *P* = 0.024), high-density lipoprotein (mg/l; *P* = 0.005), and triglycerides (mg/dl; *P* = 0.005) compared with the control group were reported in the group supplemented with 20 mg of SiO_2_@AgNPs. Moreover, significant down regulation of albumin/globulin ratio (*P* = 0.017) and significant up regulation (*P* = 0.004) of aspartate aminotransferase (U/L) were noted in groups fed 16 and 20 mg of SiO_2_@AgNPs compared with control chickens. In contrast, no significant differences were reported for TP, albumin, alkaline phosphatase, alanine aminotransferase, total lipid, total cholesterol, low-density lipoprotein, uric acids, and creatinine among the different groups. Regarding the oxidative/antioxidative parameters, MDA, catalase, and TAC showed no significant differences between the control and treated groups (Table 4).

**Table 4:** Effect of silver dopped silica nanoparticles (SiO_2_@AgNPs) on some serum biochemical parameters of boiler chickens at 35 of age

Items	Silica- silver nanoparticles levels (mg/kg diet)					
	0	8	16	20	SEM	*P* value
Total Protein (g/dl)	5.70	5.76	5.93	5.97	0.06	0.108
Albumin (g/dl)	3.03	3.15	2.83	3.00	0.07	0.067
Globulin (g/dl)	2.67^b^	2.79^ab^	2.83^ab^	2.97^a^	0.04	0.024
Albumin/ Globulin ratio	1.14^a^	1.11^a^	1.00^b^	1.01^b^	0.02	0.017
Alkaline phosphatase (μ/L)	1113.30	1115.70	1113.00	1110.30	0.56	0.271
Alanine aminotransferase (U/L)	64.00	63.30	62.00	63.00	0.41	0.161
Aspartate aminotransferase (U/L)	54.67^c^	54.99^bc^	56.67^ab^	58.00^a^	0.43	0.004
Total lipids (mg/dl)	453.33	439.01	439.33	440.00	7.80	0.129
Total Cholesterol (mg/l)	212.67	214.12	215.00	211.33	1.17	0.775
Low density lipoprotein (mg/l)	42.00	41.15	38.67	39.67	0.551	0.183
High density lipoprotein (mg/l)	96.00^bc^	94.76^c^	99.67^ab^	102.00^a^	0.90	0.005
Triglycerides (mg/dl)	182.67^b^	187.01^b^	185.67^b^	193.00^a^	1.17	0.008
Uric acid (mg/dl)	4.33	4.13	4.50	4.43	0.15	0.687
Creatinine (mg/dl)	1.23	1.19	1.17	1.20	0.02	0.816
Catalase (U/L)	366.67	365.18	363.33	360.00	2.49	0.857
Malondialdehyde (nmol/ ml)	11.00	11.11	11.33	11.67	0.23	0.827
Total antioxidant capacity (mg/dl)	1.41	1.42	1.41	1.41	0.01	0.634

^a-^^c^ Means in the same row having different letters are significantly different

### Evaluation of mRNA expression of IL-1β and TNF-α in muscle tissues

**Fig. 1:**
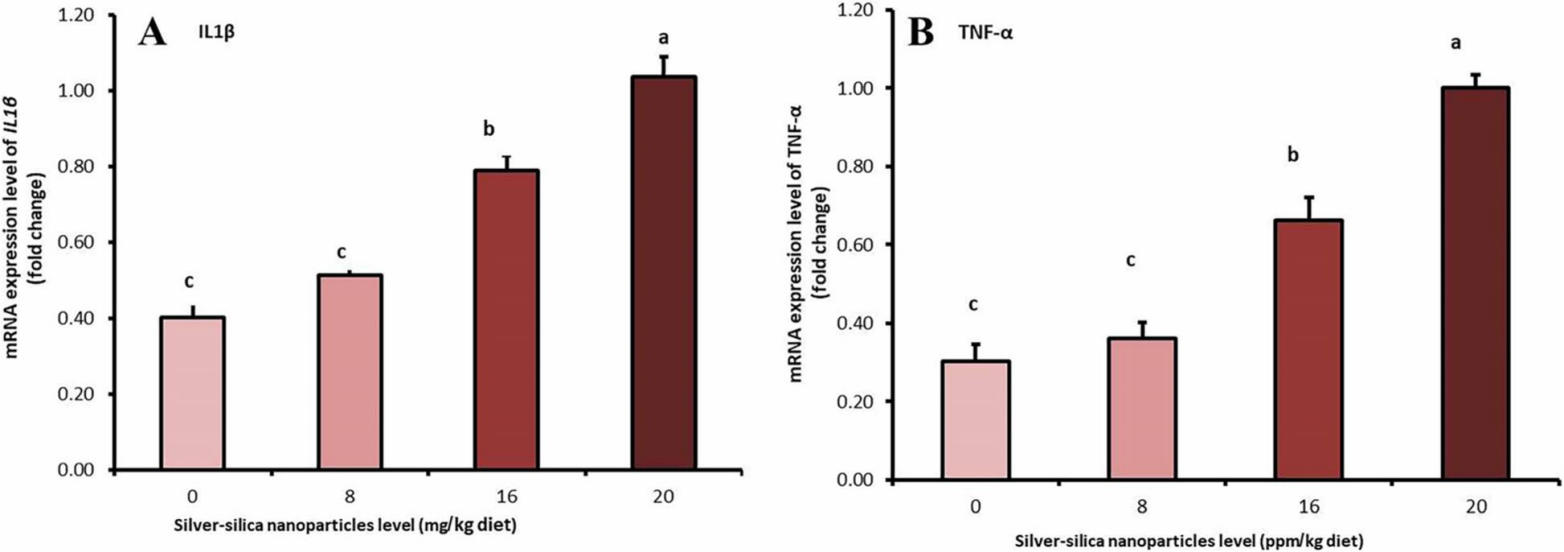
Effect of SiO_2_@AgNPs on mRNA expression of IL1β and TNF-α in muscle tissues. Groups having different letters are significantly different at (*P* < 0.05)

As presented in Fig. 1, the mRNA expression of IL-1β and TNF-α in breast muscle of control and treated chickens showed significant increases (*P* < 0.05) in the groups supplemented with 16 and 20 mg of SiO_2_@AgNPs compared with the control group.

### Evaluation of the histopathology of different tissues

#### Liver

**Fig. 2:**
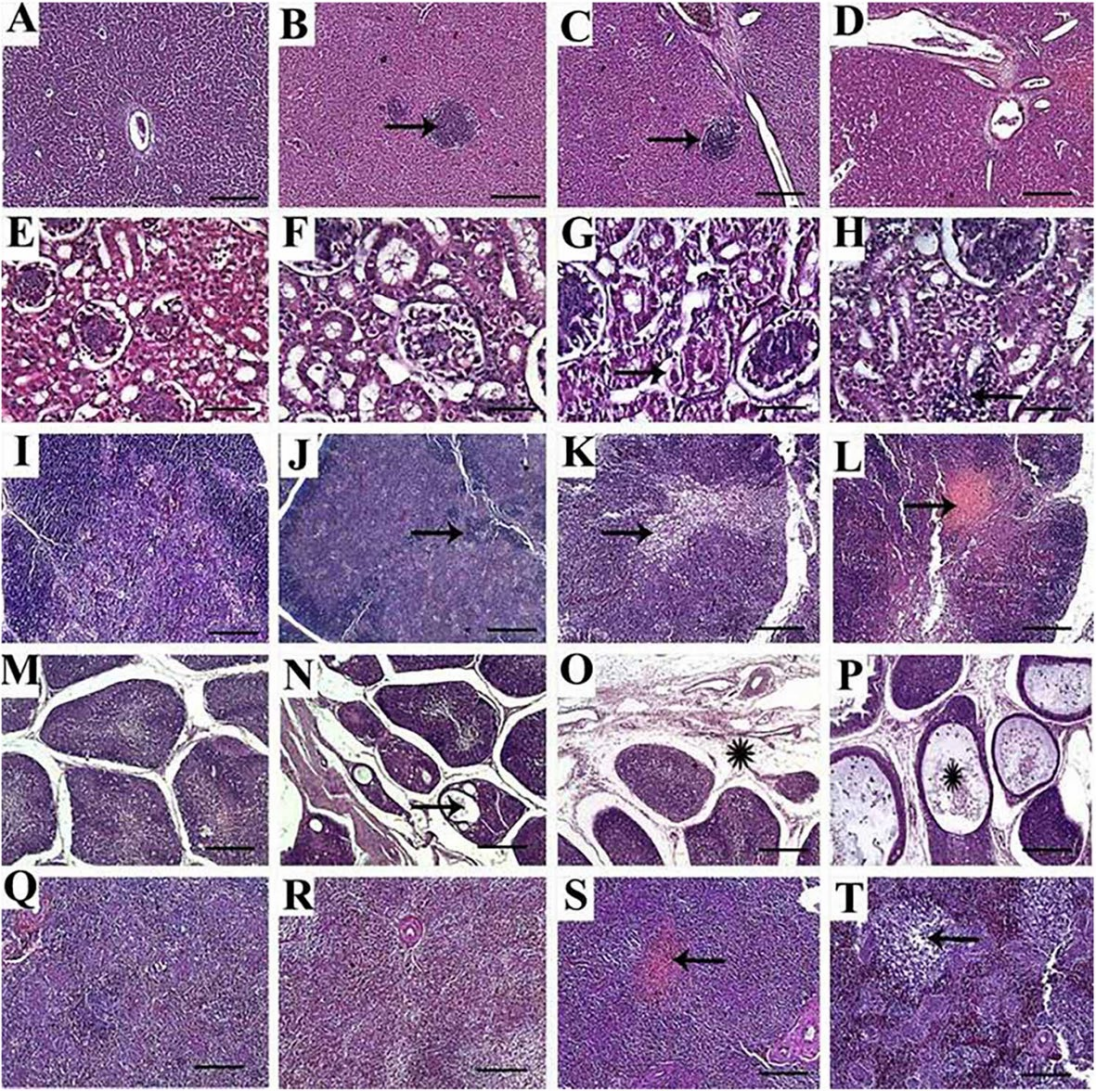
Representative photomicrographs for liver (**A**-**D**), kidney (**E**–**H**), thymus (**I**-**L**), bursa of Fabricius (**M**-**P**) and spleen (**Q**-**T**) of broiler chickens treated with different levels of SiO_2_@AgNPs for 35 days; H&E stain; bar = 100 μm for liver, thymus, bursa, spleen; and bar = 100 μm for kidney. Chickens from control group (**A**, **E**, **I**, **M**, **Q**); chicken treated with 8 mg of SiO_2_@AgNPs (**B**, **F**, **J**, **N**, **R**); chicken treated with 16 mg of SiO_2_@AgNPs (C, G, K, O, S); chicken treated with 20 mg of SiO_2_@AgNPs (**D**, **H**, **L**, **P**, **T**) showed: healthy normal liver tissue with no specific lesion (**A**); infrequent multifocal accumulations of mononuclear cells (arrows) (**B**); infrequent multifocal accumulations of mononuclear cells (arrow) and thickening of the periportal fibrous tissues (**C**); periportal fibrosis and newly formed bile ductules (**D**); normal histologic structure of renal tubules, renal epithelium, and glomerulus (**E**–**F**); mild to moderate degeneration and vacuolization of renal epithelium (arrow) (**G**); interstitial infiltration of mononuclear inflammatory cells (arrow) (**H**); normal histologic structure with normal intensity of medullary and cortical thymocytes and distinct corticomedullary junction (**I**); marked loss of cortical basophilic thymocytes (arrow) (**J**); marked reduction of cortical basophilic thymocytes and depletion in medullary thymocytes (arrow) (**K**); marked reduction of cortical basophilic thymocytes with depletion and necrosis of medullary thymocytes (arrow) (**L**); normal histologic structure of bursa with normal size and number of follicles and normal intensity of lymphocytic populations within medulla and cortex (**M**); reduced size and number of follicles, reduced medullary cell populations, and frequent cystic structure formation in some bursal follicle (arrow) (**N**); atrophy of the most of bursal follicles with abundance of interfollicular edema and inflammatory filtrates (astrisk) (**O**); interfollicular edema and inflammatory filtrates, and the most of follicles are atrophied and contain large cystic structure that contains remnants of tissue debris (astrisk) (**P**); normal histologic limits of white and red pulps and lymphoid follicles (**Q**); reduced size of lymphoid follicles (**R**); complete absence of lymphoid follicles with focal area of necrosis (arrow) (**S**); complete absence of lymphoid follicles and focal lymphoid depletion (arrow) (**T**)

Histopathological examination of chickens in the control group revealed healthy liver tissue with normal histological limits of hepatic lobules, central veins, and portal triads, with no specific lesions (Fig. 2A). Meanwhile, chickens supplemented with SiO_2_@AgNPs at levels of 8 mg (Fig. 2B) and 16 mg (Fig. 2C) showed infrequent hydropic vacuolization and multifocal accumulation of mononuclear cells. In addition, thickening of the periportal fibrous tissues was noted in chicks provided with SiO_2_@AgNPs at 16 mg. Moreover, chickens treated with 20 mg of SiO_2_@AgNPs showed frequent hepatocyte vacuolization, multifocal accumulation of mononuclear inflammatory cells, multifocal areas of coagulative necrosis, periportal fibrosis, and newly formed bile ductules (Fig. 2D).

#### Kidney

Histopathological examination of renal tissues revealed that kidney of the control chickens and those treated with 8 mg of SiO_2_@AgNPs showed similar nearly normal histological structures of renal tubules, renal epithelium, and glomerulus (Fig. 2E and F). However, renal tissues from chicks provided with 16 mg of SiO_2_@AgNPs showed mild to moderate degeneration and vacuolization of renal epithelium with occasional intertubular hemorrhage (Fig. 2G). Furthermore, kidney from chickens supplemented with SiO_2_@AgNPs at 20 mg showed moderate vacuolization and degeneration of renal epithelium, with interstitial infiltration of mononuclear inflammatory cells (Fig. 2H).

#### Thymus

Thymus tissues from the control group showed a normal histological structure with normal intensity of medullary and cortical thymocytes and distinct corticomedullary junctions (Fig. 2I). However, chicks provided with 8 mg of SiO_2_@AgNPs showed congestion, edema, and marked loss of cortical basophilic thymocytes (Fig. 2J). Moreover, chicks supplemented with 16 mg of SiO_2_@AgNPs showed marked reduction of cortical basophilic thymocytes and depletion of medullary thymocytes (Fig. 2K). Moreover, the thymus of chicks supplemented with 20 mg of SiO_2_@AgNPs showed marked edema and reduction of cortical basophilic thymocytes with depletion and necrosis of medullary thymocytes (Fig. 2L).

#### Bursa of Fabricius

The control group showed a normal histological structure of the bursa with normal size and number of follicles, prominent corticomedullary junction, and normal intensity of lymphocytic populations within the medulla and cortex (Fig. 2M). However, chickens that received 8 mg of SiO_2_@AgNPs showed reduced size and number of follicles, reduced medullary cell populations, and frequent cystic structure formation in some bursal follicles (Fig. 2N). Moreover, bursa from chickens treated with 16 mg of SiO_2_@AgNPs showed atrophy of most of the bursal follicles with an abundance of interfollicular edema and inflammatory filtrates (Fig. 2O). However, the bursa from chickens provided with 20 mg of SiO_2_@AgNPs exhibited interfollicular edema and inflammatory filtrates, where most of the follicles were atrophied and featured a single large cystic structure containing tissue debris (Fig. 2P).

#### Spleen

Splenic tissues from control chickens showed normal histological limits of white and red pulps and lymphoid follicles (Fig. 2Q). However, chickens that received the smaller dose of SiO_2_@AgNPs (8 mg) showed a reduced size of lymphoid follicles and multifocal lymphoid depletion (Fig. 2R). However, chickens treated with 16 mg showed the complete absence of lymphoid follicles (Fig. 2S). Moreover, chickens treated with 20 mg of SiO_2_@AgNPs showed the complete absence of lymphoid follicles and a multifocal area of necrosis (Fig. 2T).

### Evaluation of immunohistochemical expression of CD45

#### Liver

**Fig. 3:**
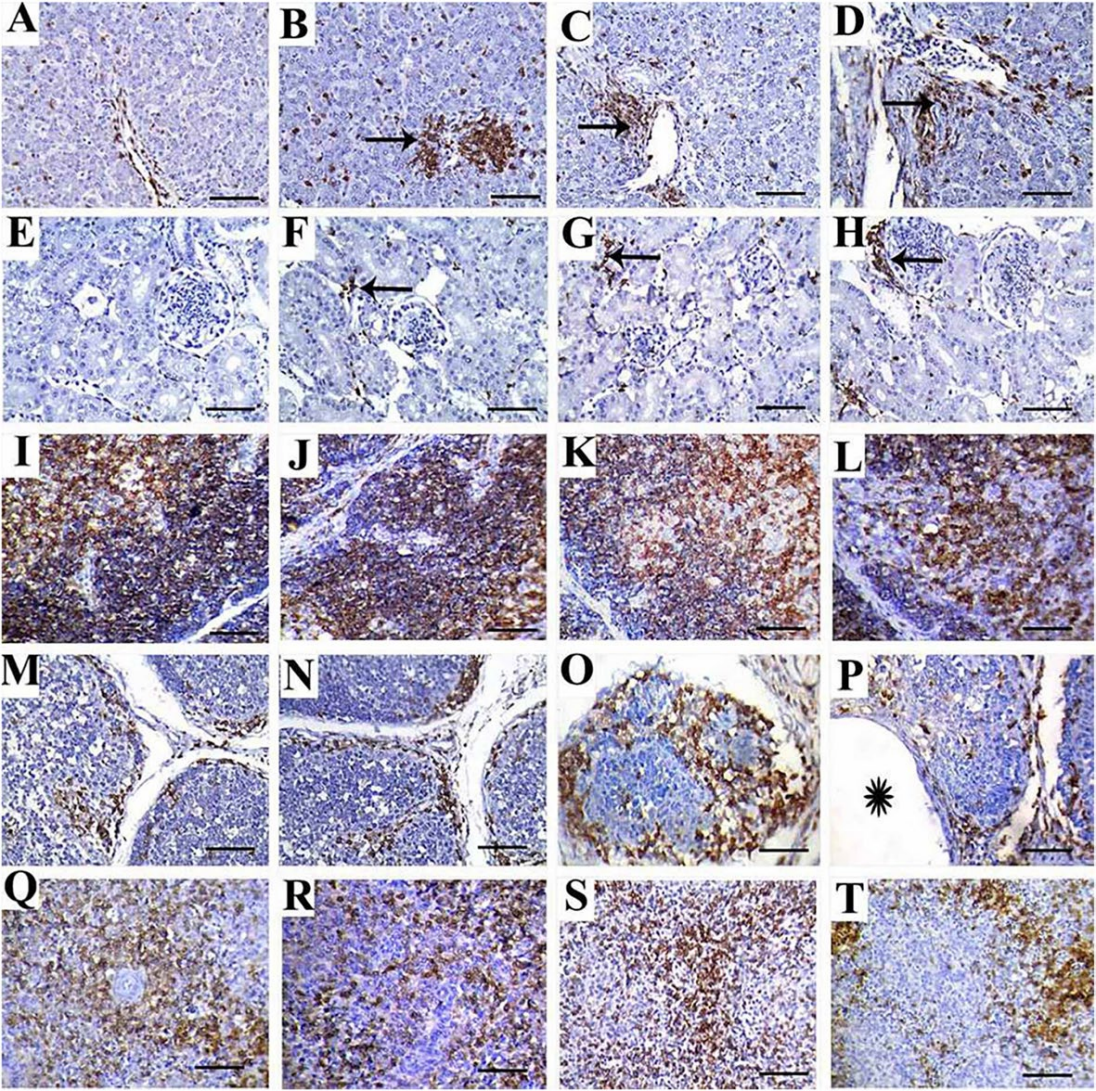
Representative photomicrographs for liver (**A**-**D**), kidney (**E**–**H**), thymus (**I**-**L**), bursa of Fabricius (**M**-**P**) and spleen (**Q**-**T**) of broiler chickens treated with different levels of SiO_2_@AgNPs for 35 days; CD45 immunostaining; bar = 50 μm. Chickens from control group (**A**, **E**, **I**, **M**, **Q**); chicken treated with 8 mg of SiO_2_@AgNPs (**B**, **F**, **J**, **N**, **R**); chicken treated with 16 mg of SiO_2_@AgNPs (**C**, **G**, **K**, **O**, **S**); chicken treated with 20 mg of SiO_2_@AgNPs (**D**, **H**, **L**, **P**, **T**) showed: positive immune expression in form of frequent individual infiltration of leukocytes between hepatocytes (**A**); multifocal areas of mononuclear leukocytic aggregations (arrow) (**B**); positive immunoexpression of the periportal leukocytic aggregates (arrows) (**C**, **D**); negative immune expression against CD45 (**E**); infrequent interlobular positively stained leukocytes (arrows) (**F**–**H**); positive immune staining against CD45 in medullary thymocytes (I); mild reduction in the immunostaining of medullary thymocytes (**J**); marked reduced intensity of immune expression of medullary thymocytes (**K**, **L**); mild to moderate immunoexpression of CD45 in the periphery of bursal follicular (**M**, **N**); marked CD45 expression within the atrophied follicle (**O**) and follicles with cystic structure (astrisk) (**P**); marked immunoexpression of CD45 at the periarterial lymphoid sheath (PALS) (**Q**, **R**); mild to moderate CD45 immunoexpression within PALS (**S**, **T**)

The immune expression of CD45 in liver tissues of control chickens revealed frequent individual infiltration of leukocytes between hepatocytes (Fig. 3A). However, chickens treated with 8 mg of SiO_2_@AgNPs showed multifocal areas of mononuclear leukocytic aggregations (Fig. 3B). Moreover, chickens supplemented with 16 and 20 mg of SiO_2_@AgNPs revealed immunoexpression in the form of frequent intralobular aggregates of mononuclear leukocytes in addition to periportal leukocytic aggregations (Fig. 3C and D).

#### Kidney

Negative immune expression of CD45 was observed in renal tissues of control chickens (Fig. 3E). However, chickens treated with 8, 16, and 20 mg of SiO_2_@AgNPs showed frequent intertubular and periglomerular positively stained leukocytes (Fig. 3F–H).

#### Thymus

In thymus, positive immune staining of CD45 was evident in the medullary thymocytes of control chickens (Fig. 3I). However, mild reduction in the immunostaining of medullary thymocytes was noted in chickens supplemented with SiO_2_@AgNPs at 8 mg (Fig. 3J). Moreover, markedly reduced intensity of immune expression was reported in medullary thymocytes of chickens provided with SiO_2_@AgNPs at doses of 16 and 20 mg (Fig. 3K and L).

#### Bursa of Fabricius

Mild to moderate positive immunoreactivity against CD45 was expressed within follicles and in interfollicular areas of control chickens and those treated with SiO_2_@AgNPs at 8 mg (Fig. 3M and N). However, more abundant immunoexpression was noted in atrophied follicles and inflamed widened interfollicular spaces of chickens receiving SiO_2_@AgNPs at doses of 16 and 20 mg (Fig. 3O and P, respectively).

#### Spleen

The immuneexpression against CD45 was noted in the spleen tissue of both control and Si-AgNP-treated chickens at the periarterial lymphoid sheath (PALS), the site where T lymphocytes aggregate in the spleen. According to the distribution of CD45 immune expression, there was pronounced T-lymphocyte aggregation in control chickens as well as those receiving 8 mg of SiO_2_@AgNPs (Fig. 3Q and R). In contrast, chickens provided with 12 and 16 mg of SiO_2_@AgNPs exhibited marked reductions in this immune expression, indicating different degrees of immune depletion in those groups (Fig. 3S and T).

### Evaluation of the ultrastructural morphology of muscle tissues

**Fig. 4:**
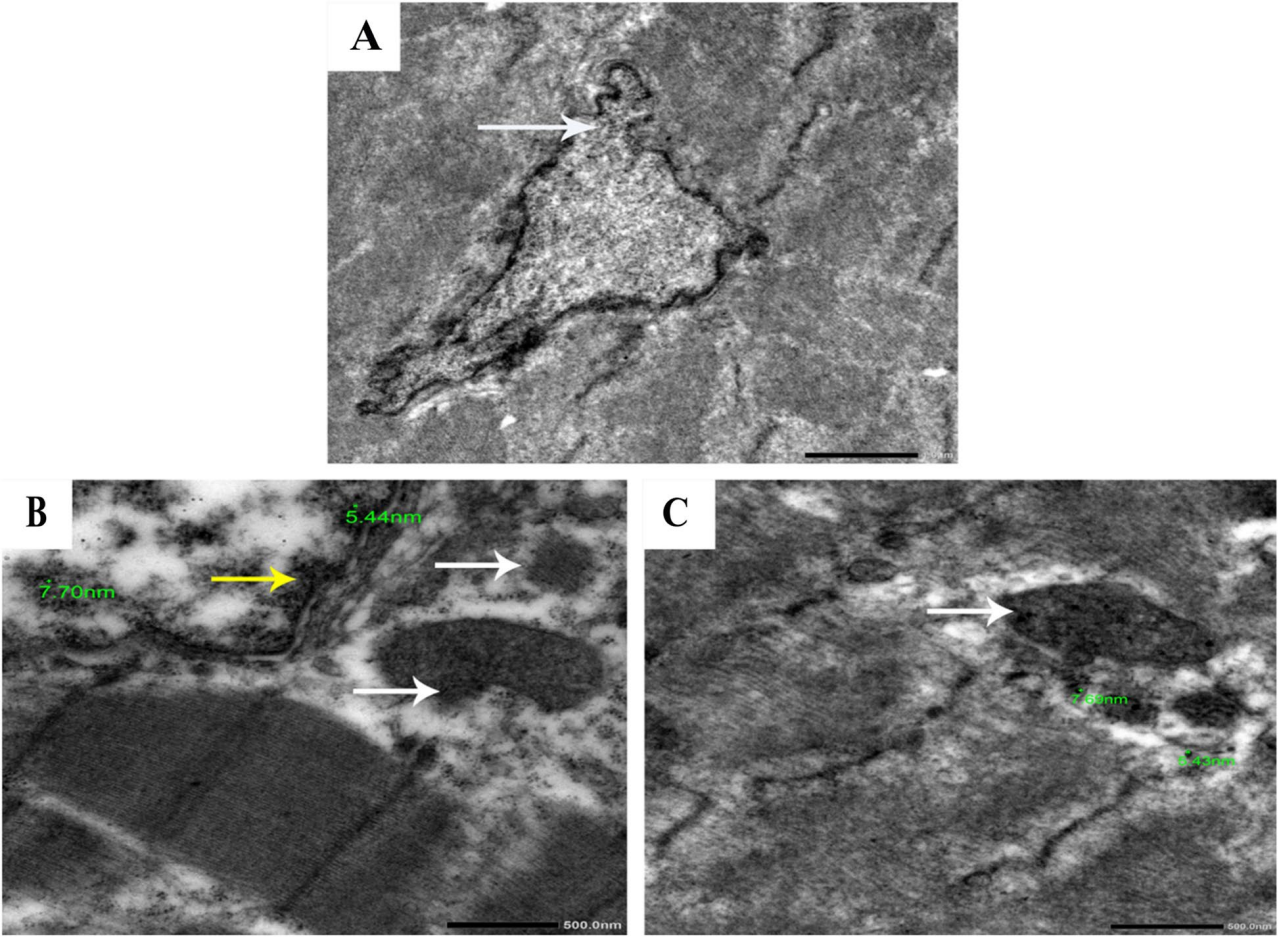
Representative photomicrographs for ultrastructure morphology of muscle tissues from broiler chickens treated with different levels of Silica- silver nanoparticles (SiO_2_@AgNPs) for 35 days: (**A**) Chickens treated with 8 mg of SiO_2_@AgNPs showed irregular nucleus and abnormal nuclear envelope (arrow), with disrupted filament and Z band. **B** Chicken supplemented with 16 mg of SiO_2_@AgNPs showed disintegrated nuclear chromatin, mild cytoplasmic vacuolization, irregular mitochondria (white arrows), and aggregations of SiO_2_@AgNPs deposits within the nuclear chromatin nearby the nuclear envelope (yellow arrows). **C** Chicken supplemented with 20 mg of SiO_2_@AgNPs showed aggregations of SiO_2_@AgNPs deposits within the internal membrane of mitochondrial cristae (white arrow)

By comparison with the control sample, TEM was used to determine the presence or absence of SiO_2_@AgNPs in chicken breast muscle and, if present, to localize them. In the control group, typical muscle composition was observed, including regular nucleus, nuclear membrane, spherical or ovoid mitochondria with well-developed cristae, normal filament, and intact Z unit. Chicken muscles receiving SiO_2_@AgNPs at a dosage of 8 mg, however, displayed an unusual nucleus and abnormal nuclear membrane, distorted filament and Z band (Fig. 4A). However, chickens supplied with SiO_2_@AgNPs at 12 and 16 mg showed similar lesions identified as abnormal nuclei and nuclear membrane, mild cytoplasmic vacuolization, disintegrated chromatin, irregular mitochondria with fragmented mitochondrial cristae, and the deposition of SiO_2_@AgNP aggregations within the nuclear chromatin and around the nuclear membrane (Fig. 4B) or within the internal membrane of mitochondrial cristae (Fig. 4C).

### Evaluation of tissue residue

**Fig. 5:**
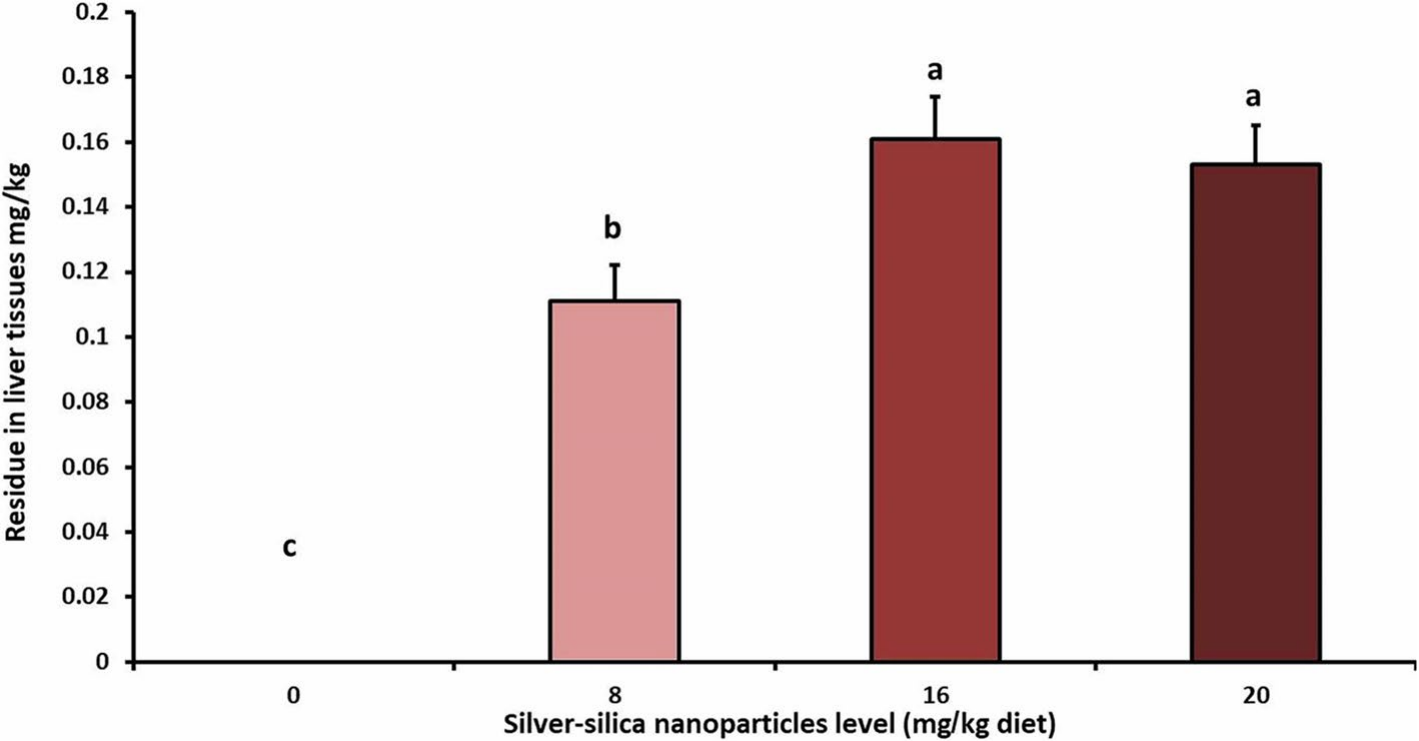
Liver residues of silver-silica nanoparticles in different treated groups (mg/kg). Groups having different letters are significantly different at (*P* < 0.05)

As shown in Fig. 5, the residual content of SiO_2_@AgNPs was significantly increased in liver tissues of treated broilers compared with that in control ones. The most prominent increase was reported in groups supplemented with 16 and 20 mg/kg diet, followed by birds treated with 8 mg/kg diet.

## Discussion

In this study, the most striking observations were the non-significant changes in growth performance parameters, oxidative parameters, and most hematological and biochemical parameters evaluated in SiO_2_@AgNP-supplemented chickens. However, the expression of genes encoding inflammatory cytokines and the ultrastructural morphology of muscle tissues as well as the histopathological and immunohistochemical findings of different tissues revealed dose-related injury in SiO_2_@AgNP-supplemented chickens. In this study, the non-significant increase in final LBW and weight gain come in agreement with findings of Hung et al. [38], who reported that no significant changes in feed consumption of rabbits supplemented with silver nanoparticles in drinking water. In contrast, [39, 40] reported significant increases in body weight of broiler chickens supplemented with AgNPs.

The hematological profile is an important indicator of the physiological or pathophysiological condition of animals [41], which reflects animals’ physiological response to various factors in the internal and external environments, such as feed and feeding [42]. In this study, a significant increase in WBC count in chickens supplemented at a dose of 20 mg/kg diet was reported. This finding agrees with our reported histopathological lesions and the significant expression of proinflammatory cytokines (IL-β1 and TNF-α) in broilers supplemented with a higher concentration of SiO_2_@AgNPs. These results suggested the proinflammatory activity of SiO_2_@AgNPs at a high dosage.

Lymphoid organ weights are commonly used to assess immunity in poultry [43]. In this study, the relative weight of spleen was decreased in SiO_2_@AgNP-supplemented chickens compared with that of control chickens. This decrease might be attributable to the lymphoid depletion associated with the increased concentration of SiO_2_@AgNPs [44]. The histopathological examination of splenic tissue confirmed this suggestion, in which we identified marked lymphoid depletion and the complete absence of lymphoid follicles with multifocal necrotic area. However, the significant increase in the relative weight of the thymus may be associated with the presence of hemorrhage and edema, as identified by the histopathological examination.

In this research, globulin and AST were significantly increased in chickens supplemented with SiO_2_@AgNPs at dose 20 mg/kg diet and doses16 and 20 mg/kg diet; respectively), which indicated hepatic injury at these doses. This was confirmed by the histopathological examination, in which vacuolization and focal necrosis were detected in chickens supplemented with 16 and 20 mg/kg diet of SiO_2_@AgNPs. The necrotic hepatocytes could permit the release of liver enzymes including AST [45]. In agreement with this, [46] reported significant increases in TP, albumin, and gamma globulin in AgNP-supplemented broilers.

Another feature evaluated in the current study is the expression of genes encoding proinflammatory cytokines in breast muscle of control and treated chickens. The higher concentrations of SiO_2_@AgNPs (16 and 20 mg/kg diet) resulted in the substantial expression of the inflammatory cytokines IL-1β and TNF-α in breast muscle tissues. Dietary exposure to silver nanoparticles could generate the intracellular ROS in mammalian cells, with the possible initiation of an inflammatory response [47–49]. The inflammatory cytokine release and the correlated production of ROS are considered as natural protective responses [49]. In this study, although significant elevations of IL-1β and TNF‐α were noted in groups supplemented with high levels of SiO_2_@AgNPs (16 and 20 mg/kg diet), serum MDA levels in these groups showed non-significant increases. This may suggest that a longer experimental period was required to produce an amplification loop between the oxidative stress and inflammatory response initiated by SiO_2_@AgNP exposure.

In this study, as the dose of SiO_2_@AgNPs increased, the residues in liver tissue increased significantly. In parallel with our results, [50] reported that the silver retention in the liver, spleen, kidney, heart, intestine, and stomach was increased upon the supply of a higher dose of silver. Our results are also supported by [51], who reported that the higher retention of AgNPs in broiler organs primarily involved the small intestine, followed by the liver tissues. In contrast, [26] concluded that the accumulation of AgNPs in rat organs was not significant in orally treated rats rather than those with intravenous injection, and asserted that the orally administered AgNPs passed through the gastrointestinal tract and were excreted via the feces without translocation in the bloodstream.

In this work, histopathological examination of the liver, kidney, thymus, bursa, and spleen from control and SiO_2_@AgNP-treated chickens (8, 16, and 20 mg) revealed moderate to severe pathological lesions with severity that correlated with the level of SiO_2_@AgNP supplementation. The reported lesions were in the form of diffuse mononuclear inflammatory infiltrates, necrotic and degenerative changes in hepatic and renal tissues, and even fibroplasia in hepatic portal triads, in addition to lymphoid cell depletion in spleen, thymus, and bursa. Also, [52] reported similar observations, where researchers noted slight necrosis and inflammatory infiltrates in hepatic tissues of broiler chickens supplemented with the maximum concentration of AgNPs. Additionally, [53] observed that the supplementation of AgNPs led to dose-dependent hepatic injuries, particularly necrosis, fibroplasia, and polymorphonuclear leukocyte proliferation. Moreover, [53] demonstrated that several areas of lymphoid depletion and hemorrhage have been identified in the bursa of Fabricius of AgNP-supplemented chickens. These effects may be due to the capacity of AgNPs to infiltrate the cells of different tissues/organs, including the liver, kidneys, and lymphoid organs, by binding to plasma proteins [54].

With a corresponding upregulation of inflammatory cytokines and ROS production, AgNPs can diminish the mitochondrial function within cells [55, 56]. Also, [57] concluded that the mitochondrial disruption caused by AgNPs was increased by increasing the concentration of AgNPs. Thus, mitochondria may also be considered targets susceptible to the cytotoxicity of AgNPs. Accordingly, [55] stated that a rat liver cell line (BRL 3A) treated with AgNPs displayed irregular mitochondrial shape and size. This discovery is in line with our ultrastructural morphology findings. The ultrastructure analysis of muscle tissues in this research revealed aggregation as singlet deposits or as dense particles in the muscle fibers. Nanoparticles were located inside the nucleus, near the nuclear membrane, within mitochondria, or deep in the cytoplasm. Specifically, the degradation of chromatin and mitochondrial cristae was observed in chickens administered 16 mg of AgNPs. Similar conclusions were drawn by [58] and [59]. They proposed that AgNPs would enter cells and concentrate in mitochondria, leading to their death. In accordance with our observations, [58, 60] and [61] identified that, the greater dosage of AgNPs, the greater the degree of accumulation of AgNP deposits.

Oxidation induced by a high dosage of SiO_2_@AgNPs will contribute to inflammatory activation and the suppression of immune functions [26, 27]. In our research, this hypothesis was formulated by assessing the relative weight and histopathologically examining lymphoid organs, estimating IgM and IgG serum levels, and evaluating CD45 immune expression. Our results revealed significant reduction in IgM and IgG in chickens supplemented with 16 and 20 mg of SiO_2_@AgNPs.

CD45 (called leukocyte common antigen, a general marker for hematopoietic cells other than erythrocytes and platelets) plays a crucial role in the immune system as a significant regulator of T- and B-cell antigen receptors [62]. To the best of our knowledge, no data concerning the effect of dietary supplementation with Ag nanoparticles on CD45 immune expression in broilers have been reported. In this study, the immune expression of CD45 was increased in SiO_2_@AgNP-supplemented chicken liver, kidney, and bursa. This upregulation may be due to the SiO_2_@AgNP-mediated inflammatory reaction, contributing to the inflow of mononuclear cells into different tissues, including the liver, kidney, and bursa. In contrast, the cortical thymocytes and spleen PALS revealed decreased expression of CD45; this result may be due to the adverse impact of a higher dose of SiO_2_@AgNPs on birds’ immune response, as mentioned above [26, 27].

## Conclusion

Based on the current findings, the dietary application of SiO_2_@AgNPs at a dose of 8 mg/kg diet or more has dose-dependent effects on the expression of genes encoding inflammatory cytokines and the ultrastructural morphology of muscle tissues as well as the histopathological and immunohistochemical findings of different tissues. Detailed study of the toxic effects of different nanomaterial has become an urgent need to avoid the potential negative effects of these materials while maximizing their use as natural alternatives in order to reduce antibiotic resistance. Studying different doses of SiO_2_@AgNPs on pathogenic bacteria in the intestine is still necessary and should be followed in future studies.

## Supplementary Information

**Additional file 1.**

## Data Availability

All data generated or analyzed during this study are included in this published article and its supplementary information files.
