# Supplementary material for: Silver-Silica nanoparticles induced dose-dependent modulation of histopathological, immunohistochemical, ultrastructural, proinflammatory, and immune status of broiler chickens
Source: BMC Vet Res. 2022 Oct 4;18:365. doi: 10.1186/s12917-022-03459-2 (PMC9531355; doi:10.1186/s12917-022-03459-2)
Supplement: Supplementary file 1 — Additional file 1. [file 12917_2022_3459_MOESM1_ESM.docx]

**Supplementary materials for preparation and characterization of silver dopped-silica nanoparticles used in study**

1. **Preparation of silver doped-silica nanoparticles (SiO_2_@AgNPs)** **in powder form**

At first, 0.5 g of starch is dissolved in 100ml of water, then, 0.25 g of sodium hydroxide is added to the starch solutuion to reach a pH; 11. To this end, solution of silver nitrate (2%w/v) is added dropwise to the previous starch solution under continuous stirring. The temperature is raised to 70^o^C. The obtain product is namely silver nanoparticles; AgNPs and coded as: (solution A).

The sol-gel synthesis is outlined as follow: the sol was prepared by mixing the precursor tetraethylorthosilicate (TEOS, 200ml) , with alcohol (400ml) and water (85ml). The mixture of ethanol-water was added dropwise in order to avoid the rapid hydrolysis of the precursor. After the complete addition of the cosolvent, the ready sol-solution is coded as (Solution B). To this end, solution B is added dropwise to solution A, under continuous stirring. Then, ammonia is added to precipitate the formed SiO_2_ and AgNPs together. The solution is subjected to filteration, followed by washing the PPT several times with excess amount of water to remove residual NH_4_OH and unreacted tetraethyl orthosilicate. The end product is silver nanoparticles dopped silica or silver-silica nanoparticles in powder form and it is coded as SiO_2_@AgNPs.

1. **Characterization of the formed silver doped silica nanoparticles (**SiO_2_@AgNPs)**.**

In order to examine the prepared sample with the TEM, sample of SiO_2_@AgNPs was deposited in on a “carbon-coated copper grid” and was left for drying at room temperature followed by characterization thru “TEM instrument JEOL 200 kV, Japan”. “Nano-Sizer SZ90, Malvern instruments Ltd., UK” was used to evaluate the “particle size” as well as “zeta potential” for the formed SiO_2_@AgNPs in its powder form. The size distribution and zeta potential of the as prepared SiO_2_@AgNPs was calculated at pH = 7 and 25°C. “Scanning Electron Microscopy; SEM; JEOL, JSM-6360LA, Japan” was utilized to illustrate the internal structure and surface morphology of SiO_2_@AgNPs. “X-ray diffraction; XRD” analysis was proceeded to check the crystallinity and the specific peaks for the formed SiO_2_@AgNPs by via “XRD sate of art “Panalytical Emperian, Turkey” pertaining CuKa radiation and is operated with power; 40 kV and a 2-theta range of 10–80.

As known, the particle shape of the resultant nanoparticles examined via TEM was carried out for the sample after placing it onto the copper coated grid. Thus, the particle does not have the tendency to agglomerate during the measurement. Hence, the size of the particles is formed with nearly small size when compared with dynamic light scattering study (DLS). The latter needs the evaluated sample to stand in the instrument for a long time in a solution. So, the particles in the solution tend to aggregate into large particles.


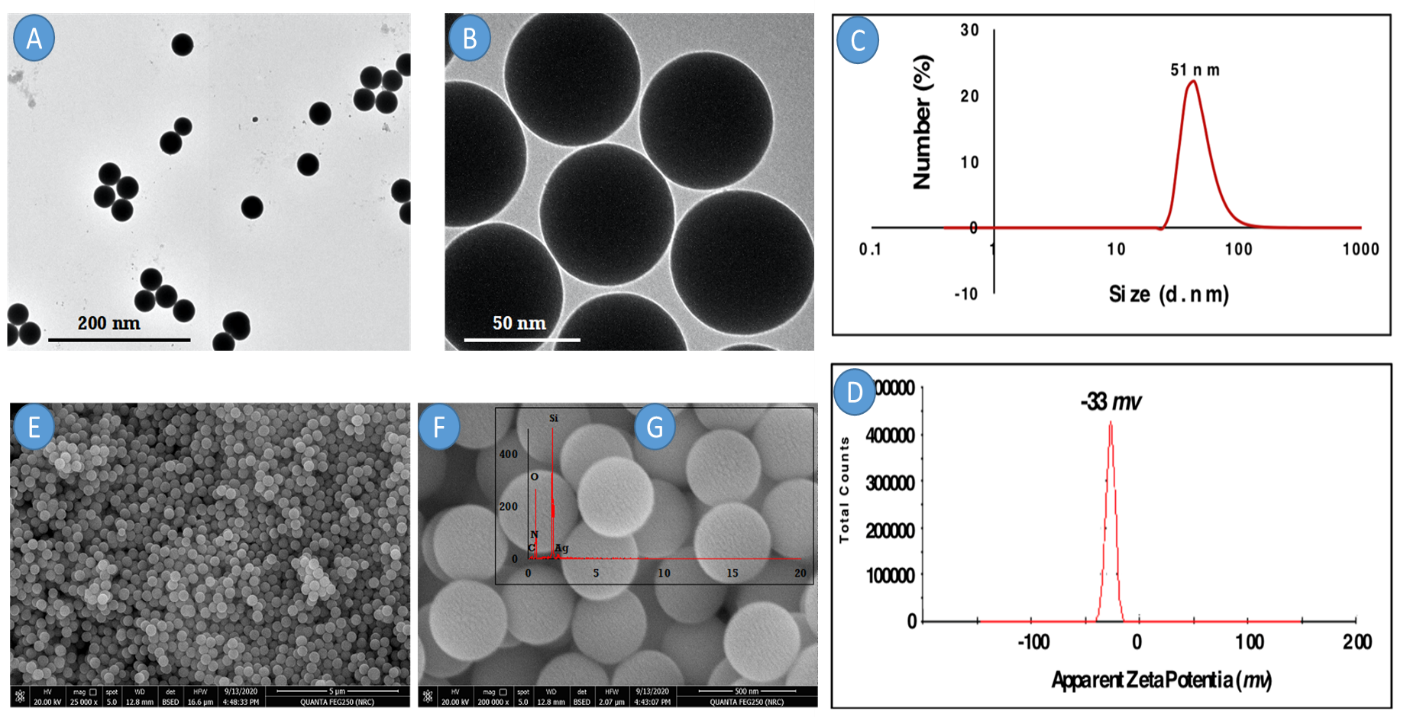


**Figure 1.** TEM of SiO2@AgNPs at (A) low magnification and (B) high magnification& (C) hydrodynamic size and (D) apparent zeta potential of SiO2@AgNPs & (E) SEM of SiO2@AgNPs at low magnification and (F) high magnification and (G) EDX of SiO2@AgNPs.

Figure 1 C) represents the hydrodynamic size of SiO_2_@AgNPs, it is revealed that the average hydrodynamic diameter of SiO_2_@AgNPs is 51 nm with the polydispersity index (PDI) below 0.24 (below 0.5) affirming that, the nanoparticles possess relatively narrow size distribution and well dispersibility. In addition, SiO2@AgNPs has negative zeta potential (-33 mv) due to the presence of stabilizing agent stabilized the resulted nanoparticles from agglomeration. Figure 1 D) shown that the zeta potential of SiO_2_@AgNPs is less than 30 mV (-33 mv) indicating the good stability of these nanoparticles in aqueous solution. The surface structure and morphology of SiO_2_@AgNPs was investigated using SEM. Figure 1 (E, F) displays the morphological structure of SiO2@AgNPs at two magnifications (25 000 x and 200000 x) respectively. SiO_2_@AgNPs as fairly uniform spherical particles with an average size of 150–250 nm. It is remarkable that the particles are formed with sufficient aggregation which could be attributed to by the polydispersity of the sample and indirectly confirms the presence of aggregate particles. Therefore, spherical SiO2@AgNPs is formed due to isotropic growth. Briefly speaking, the anisotropic structures of SiO2@AgNPs could be induced by growth rates on different directions at low temperature. On contrary, the high temperatures resulted in the production of non-spherical particles and gives rise to the severe anisotropic growth, resulting in the formation of rod-like particles with tetragonal cross section (data not shown). Moving to our discussion, the presence of AgNPs of the reactants has no considerable effect on the morphology of SiO_2_NPs and thus, the resultant nanoparticles confirm that, the morphology of SiO_2_@AgNPs produced mainly depends on the reaction temperature. Moving to the discussion of the onset image (Figure 1 G) (elemental analysis of the scanned sample *via* EDX), it is remarkable that the sample contains four elements; carbon, oxygen, silicon and silver. The presence of carbon and oxygen are attributed to the existence of natural polymer; starch and oxygen connected to silica nanoparticles. On the other hand, the existence of Si and Ag affirming the formation of SiO_2_@AgNPs.
